# Diffuse Large B-cell Lymphoma of the Tibia in a Patient With Longstanding Seropositive Rheumatoid Arthritis on Methotrexate

**DOI:** 10.7759/cureus.23232

**Published:** 2022-03-16

**Authors:** Mansi Oberoi, Swaminathan Perinkulam Sathyanarayanan, Jacky Akther, Hamza Tantoush

**Affiliations:** 1 Internal Medicine, University of South Dakota Sanford School of Medicine, Sioux Falls, USA; 2 Pathology, University of South Dakota Sanford School of Medicine, Sioux Falls, USA

**Keywords:** lymphoproliferative disease, diffuse large b-cell lymphoma, methotrexate, lymphoma, rheumatoid arthritis

## Abstract

There has been an observance of increased occurrence of malignant lymphomas in patients with rheumatoid arthritis (RA). The increased risk of lymphoproliferative disorders has been linked to the severity of RA disease activity, the use of disease-modifying agents like methotrexate and certain genetic links between RA and lymphomas. This article outlines the case of an 88-year-old gentleman with a 13-year history of seropositive rheumatoid arthritis on methotrexate who presented with ankle pain and was subsequently found to have diffuse large B-cell lymphoma on further workup.

## Introduction

Rheumatoid arthritis (RA) is a chronic inflammatory condition of the joints associated with significant morbidity and functional disability. Lymphoproliferative disorders (LPDs), especially lymphomas, have been known to occur with increased frequency in patients with RA, in both those who are treated with methotrexate and those who have never been exposed to methotrexate or any other immunosuppressive agent. Longstanding, active disease is the major risk factor for lymphoma development in patients with RA. The incidence increases as active RA persists and directly correlates with the severity of disease activity, reflecting the role of chronic inflammation in the underlying pathology [1,2]. 

## Case presentation

An 88-year-old gentleman with a past medical history significant for seropositive erosive rheumatoid arthritis, osteoarthritis, osteopenia, prostate cancer status post prostatectomy, squamous cell carcinoma and basal cell carcinoma of the skin, chronic kidney disease stage 3 and secondary hyperparathyroidism presented to the rheumatology clinic with progressively worsening right ankle swelling and pain. The pain was aggravated on movement and relieved by rest. He did not have any pain in other joints, joint stiffness, recent trauma or falls. He denied hair loss, dry eyes, dry mouth, oral or nasal ulcers, difficulty swallowing, chest pain, shortness of breath, skin rash, weight changes, fevers, chills, altered bowel or bladder habits. His rheumatoid arthritis was diagnosed 13 years ago. It was well controlled on methotrexate 12.5 mg once weekly however, the patient noted not having taken the medication for the last three weeks. Physical examination was remarkable for swelling and tenderness of the right ankle associated with decreased active and passive range of motion. Examination of other joints was unremarkable. Please refer to Table 1 for laboratory investigations. 

**Table 1:** Laboratory investigations

COMPLETE BLOOD COUNT WITH DIFFERENTIAL	VALUES
Leukocyte count (ref 4 to 11 k/uL)	7.5
Hemoglobin (ref 13 to 17 g/dL)	13.9
Platelets (ref 150 to 450 k/uL)	265
Neutrophil % (ref 40 to 60%)	84
Lymphocytes (ref 20 to 40%)	12
Monocytes (2 to 8%)	4
Eosinophils (1 to 4%)	0
Basophils (0.5 to 1%)	0
CHEMISTRY PANEL	
Sodium (ref 136 to 145 mEq/L)	137
Potassium (ref 3.5 to 5 mEq/L)	4.3
Chloride (ref 95 to 105 mEq/L)	104
Bicarbonate (ref 22 to 28 mEq/L)	25
Blood Urea Nitrogen (ref 5 to 20 mg/dL)	36
Creatinine (ref 0.6 to 1.1 mg/dL)	1.59
Calcium (ref 8.5 to 10.5 mg/dL)	10.9
Erythrocyte Sedimentation rate (0 to 30 mm/hr)	15
C-reactive protein (ref <5 mg/dL)	5.9
Rheumatoid factor (ref <13 IU/mL)	45.2

X-ray of the ankle did not reveal any fractures, erosions or calcium pyrophosphate deposition. There was cortical thickening of the distal fibula. Due to medication non-compliance, the pain was thought to be due to RA flare, so he was put on a steroid taper and his methotrexate was resumed. With no improvement in his symptoms, a therapeutic joint aspiration was performed two weeks later and he was given intra-articular steroid injection.

**Figure 1:**
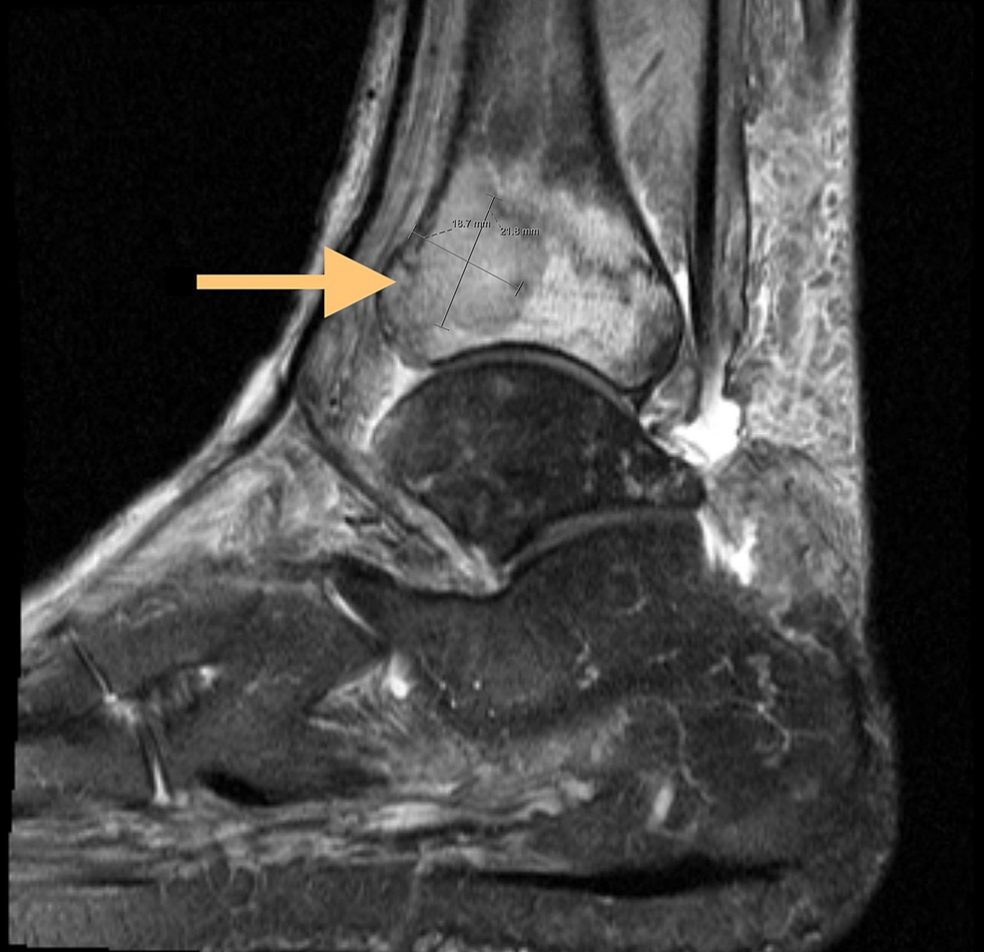
Non-displaced osteoporotic fracture of the distal tibial plafond with an abnormal area of signaling in the anterior lateral tibial plafond measuring 1.8 cm x 2.1 cm

**Figure 2:**
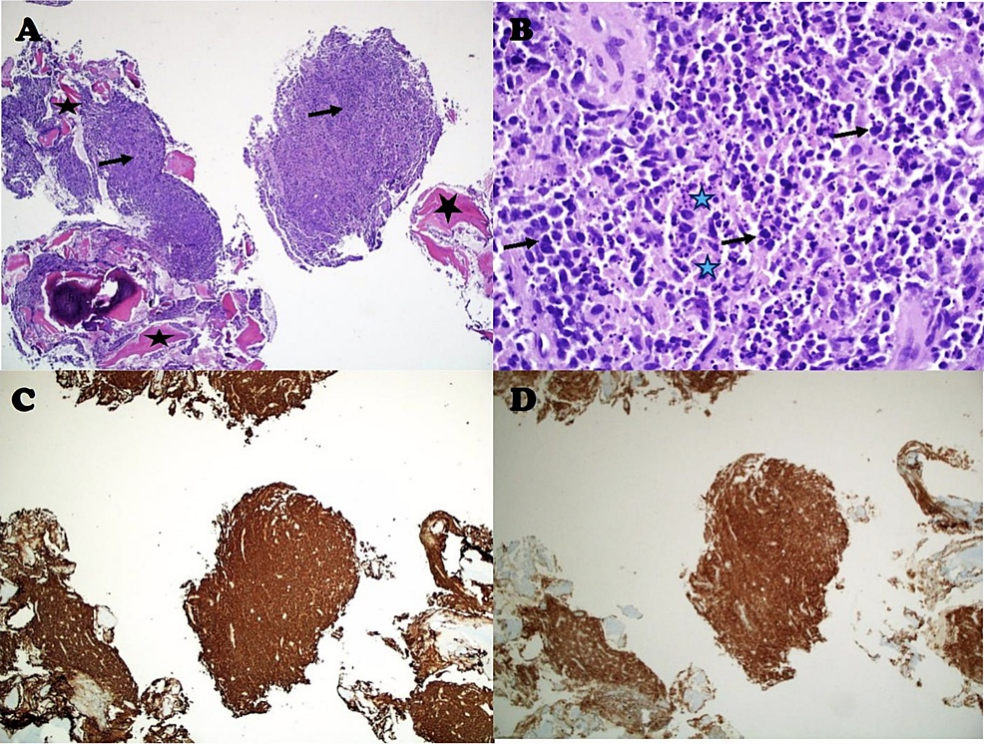
A) H&E stain of the tibial mass at 4x B) enlarged view of A, H&E stain at 40X showing bone (black star) infiltrated by large, hyperchromatic malignant cells (arrow), with abundant apoptotic bodies and necrosis (blue star) in the background. C) CD20 immunohistochemical stain, showing diffuse positivity (brown colouration) within the malignant cells, confirming B-cell origin. D) CD10 immunohistochemical stain, showing diffuse positivity (brown colouration) within the malignant cells, confirming germinal center origin.

**Figure 3:**
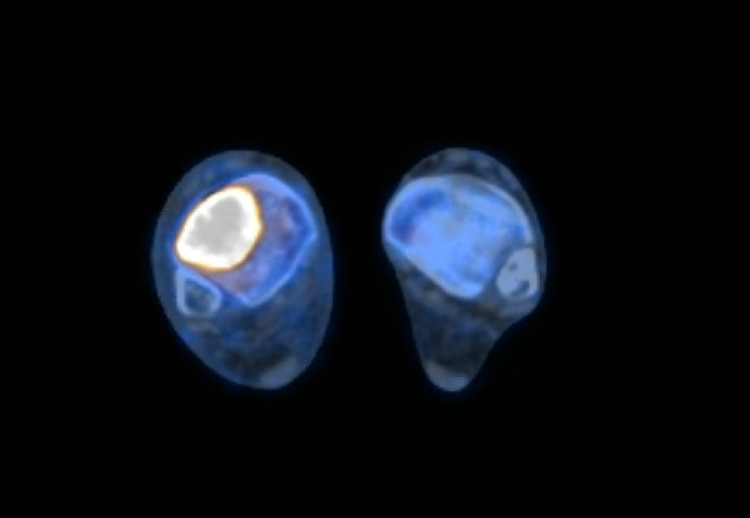
PET CT demonstrating the FDG avid area in the distal right tibia measuring approximately 4.1 x 3.4 cm with a maximum SUV (Standardized Uptake Value) of 37.1 FDG: fluorodeoxyglucose

With persistent ankle pain, a Magnetic Resonance Image (MRI) of the ankle without contrast was obtained which revealed a nondisplaced osteoporotic fracture of the distal right tibial plafond with intra-articular extension along the lateral margin. It also revealed an expansile lytic lesion in the area of fracture measuring 1.8 x 2.1 cm (Figure 1). His colchicine was discontinued, and he was referred to orthopedics. He underwent a Computed Tomography (CT) guided bone biopsy which revealed diffuse large B-cell lymphoma of germinal center cell origin (Figure 2). The cells were positive for CD45, PAX5, CD20, CD21, CD10, BCL6, MUM1 and MYC and negative for cyclin D1, PSA, pankeratin and BCL2 which was consistent with the histopathological diagnosis. He subsequently underwent a Positron Emission Tomography (PET) scan that revealed fluorodeoxyglucose (FDG) avid lytic lesion in the distal right tibia corresponding to the patient's biopsy-proven lymphoma with no evidence of disease at any other site which was consistent with Stage 1E disease (Figure 3).

He was referred to oncology for further management. There was no definitive consensus on whether his lymphoma was due to methotrexate use or due to long-standing RA. His methotrexate was discontinued. Due to his advanced age and medical comorbidities, he was not a good candidate for systemic therapy, so he was referred for definitive external beam radiation therapy. A bone marrow biopsy was not performed given his normal cell counts. He completed his radiation therapy with a total dose of 5000 cGy to the right tibia in 20 divided cycles. He tolerated the radiation well with significant improvement in pain and swelling. He continues to follow up with medical oncology every three months. Imaging following radiation treatment was not performed because further systemic therapy was not planned as per the patient’s request. During the subsequent follow-up visits (1.5 years into diagnosis), he had no symptoms to suggest disease progression.

## Discussion

RA is associated with an increased risk of developing various comorbidities including cardiovascular diseases, serious infections and malignancies. However, in recent years, there has been a dramatic improvement in clinical outcomes of patients with RA with the use of disease-modifying antirheumatic drugs (DMARDs) and biologic agents [3].

RA patients have an increased risk of development of lung cancers and lymphomas as compared to the general population. Lymphoma carries a hazard ratio (HR) of 1.6 to 2.46 in patients with RA which is much higher than other malignancies [4,5]. Non‐Hodgkin lymphomas are more common than Hodgkin’s lymphomas, of which diffuse large B cell lymphoma (DLBCL) is the most frequent subtype. Amongst the subtypes of DLBCL, the more aggressive non‐germinal center B-cell-like is more common, suggesting initial involvement of activated B cells [6]. Various mechanisms have been proposed for the association between RA and lymphoma development. Of these, three major theories include immune stimulation triggered by severe chronic inflammation, iatrogenic immunosuppression allowing immunogenic tumors to develop, and a common genetic predisposition [7].

Disease activity and risk of lymphoma

Immune stimulation triggered by severe chronic inflammation in RA may play a key role in lymphoma development. Those in the top 20th percentile of disease activity have a markedly heightened risk of developing lymphoma. Physicians should be watchful for lymphoma development in patients with RA who have high cumulative disease activity, or a Disease Activity Score - 28 for RA > 5.7 [7]. No biomarker has been identified to predict future lymphoma risk in RA patients. For example, the presence of anti-cyclic citrullinated protein IgG antibodies, which is typically associated with poor clinical outcome and radiological progression of RA, is not associated with increased lymphoma risk among RA patients [8].

Immunosuppressant use-related risk

There has been a longstanding concern that treatment for RA itself may increase the risk further, particularly treatment with DMARDs. However, immunosuppressant use is widely believed to be a confounding factor as the severity of disease activity largely correlates with their use [2].

The World Health Organization (WHO) classifies methotrexate-associated lymphoproliferative disorders (MTX-LPDs) as “other iatrogenic immunodeficiency-associated LPDs” [9]. Methotrexate can have mixed effects on lymphoma development. It may decrease the risk via decreased immunologic activity or may promote the development of some lymphomas through immunosuppression, leading to an overall null impact on incidence [7]. There are several histologic types of MTX-LPDs, of which diffuse large B-cell lymphoma (DLBCL) and classic Hodgkin lymphoma (CHL) are more common, accounting for 40% to 50% and 10% to 30% of all neoplastic lesions arising in MTX-LPDs, respectively. Interestingly, MTX-LPDs are known to often regress on MTX withdrawal [10].

On the other hand, oral corticosteroids use in the initial years of RA diagnosis was associated with a reduced risk of development of lymphomas (HR 0.5 [95% CI 0.3-0.9]) compared to those who were not initiated on steroids [5]. There is not much evidence for the association between sulfasalazine and hydroxychloroquine use with the development of malignancies, and neither of them is significant immunosuppressive DMARDs. However, few other less commonly used DMARDs including cyclosporine and azathioprine have been reported to be associated with an increased risk of malignancy [2].

Genetic risk

The increased lymphoma risk in RA might reflect a shared susceptibility or risk factors common to both RA and malignant lymphoma [11]. Single nucleotide polymorphisms involving genes like MMEL1 and CASP10 have been noted in both RA and lymphomas like DLBCL and chronic lymphocytic lymphomas respectively [12].

Treatment

To date, there are no strict guidelines for the treatment of RA-associated LPDs. However, some of the options which might be beneficial include withdrawal of immunosuppressant therapy, use of rituximab monotherapy which has anti-lymphoma and anti-autoimmune activity, chemotherapy, and T-cell immunotherapy (Epstein Barr virus-specific cytotoxic T lymphocytes [CTL]) [13]. 

## Conclusions

There is an increased risk of lymphoproliferative diseases in patients with rheumatoid arthritis. While the exact causation behind this association is elusive, the disease activity of rheumatoid arthritis and the use of medications such as methotrexate are thought to be the reasons behind this increased risk. 
